# Cognitive Decline and BPSD Are Concomitant with Autophagic and Synaptic Deficits Associated with G9a Alterations in Aged SAMP8 Mice

**DOI:** 10.3390/cells11162603

**Published:** 2022-08-21

**Authors:** Foteini Vasilopoulou, Aina Bellver-Sanchis, Júlia Companys-Alemany, Júlia Jarne-Ferrer, Alba Irisarri, Verónica Palomera-Ávalos, Celia Gonzalez-Castillo, Daniel Ortuño-Sahagún, Coral Sanfeliu, Mercè Pallàs, Christian Griñán-Ferré

**Affiliations:** 1 Department of Pharmacology and Therapeutic Chemistry, Faculty of Pharmacy and Food Sciences, Institut de Neurociències, Universitat de Barcelona, Avda. Joan XXIII, 27, 08028 Barcelona, Spain; 2 Tecnologico de Monterrey, Escuela de Medicina y Ciencias de la Salud, Zapopan 64710, Mexico; 3 Laboratorio de Neuroinmunología Molecular, Instituto de Investigación de Ciencias Biomédicas (IICB) CUCS, Universidad de Guadalajara, Guadalajara 44340, Mexico; 4 Institut d’Investigacions Biomèdiques de Barcelona (IIBB), CSIC and Institut d’Investigacions Biomèdiques August Pi i Sunyer (IDIBAPS), 08036 Barcelona, Spain

**Keywords:** BPSD, aggressivity, cognitive decline, autophagy, epigenetics, G9a, SAMP8

## Abstract

Behavioural and psychological symptoms of dementia (BPSD) are presented in 95% of Alzheimer’s Disease (AD) patients and are also associated with neurotrophin deficits. The molecular mechanisms leading to age-related diseases are still unclear; however, emerging evidence has suggested that epigenetic modulation is a key pathophysiological basis of ageing and neurodegeneration. In particular, it has been suggested that G9a methyltransferase and its repressive histone mark (H3K9me2) are important in shaping learning and memory by modulating autophagic activity and synaptic plasticity. This work deepens our understanding of the epigenetic mechanisms underlying the loss of cognitive function and BPSD in AD. For this purpose, several tasks were performed to evaluate the parameters of sociability (three-chamber test), aggressiveness (resident intruder), anxiety (elevated plus maze and open field) and memory (novel object recognition test) in mice, followed by the evaluation of epigenetic, autophagy and synaptic plasticity markers at the molecular level. The behavioural alterations presented by senescence-accelerated mice prone 8 (SAMP8) of 12 months of age compared with their senescence-accelerated mouse resistant mice (SAMR1), the healthy control strain was accompanied by age-related cognitive deficits and alterations in epigenetic markers. Increased levels of G9a are concomitant to the dysregulation of the JNK pathway in aged SAMP8, driving a failure in autophagosome formation. Furthermore, lower expression of the genes involved in the memory-consolidation process modulated by ERK was observed in the aged male SAMP8 model, suggesting the implication of G9a. In any case, two of the most important neurotrophins, namely *brain-derived neurotrophic factor (Bdnf)* and *neurotrophin-3 (NT3),* were found to be reduced, along with a decrease in the levels of dendritic branching and spine density presented by SAMP8 mice. Thus, the present study characterizes and provides information regarding the non-cognitive and cognitive states, as well as molecular alterations, in aged SAMP8, demonstrating the AD-like symptoms presented by this model. In any case, our results indicate that higher levels of G9a are associated with autophagic deficits and alterations in synaptic plasticity, which could further explain the BPSD and cognitive decline exhibited by the model.

## 1. Introduction

Advanced age is the most substantial risk factor for developing age-related neurodegenerative disorders, such as Alzheimer’s Disease (AD) and other dementias [1]. These pathological conditions manifest as progressive cognitive decline and are often accompanied by non-cognitive alterations, the so-called behavioural and psychological symptoms of dementia (BPSD). BPSD include aggression, agitation, anxiety and depressive mood, resulting in poor outcomes for AD patients [2,3,4]. To effectively address this significant health issue, it is crucial to understand the cellular and molecular mechanisms underlying the loss of cognitive function and BPSD in AD, driving their clinical manifestation.

Emerging evidence shows that epigenetics are critical in learning and memory processes, and their dysregulation has been associated with the pathogenesis of AD [5,6,7]. Of note, there are no transgenic mouse lines that model age-related AD, the most important risk factor of AD. However, in this context, the senescence-accelerated mouse 8 (SAMP8) has been proposed as a neurodegenerative mouse model to study late-onset Alzheimer’s disease (LOAD) associated with ageing [8,9,10,11]. At the same time, previous studies of our group demonstrated altered epigenetic regulation in AD mouse models, including the SAMP8 model, such as DNA methylation, hydroxymethylation, histone acetylation and miRNA modifications [12,13,14,15]. Additionally, histone modifications can repress or activate genes involved in learning-dependent synaptic plasticity, also demonstrating a role in cognition [6]. In this line of evidence, histone methylation has gained attention in the AD field [16]. Remarkably, we and others have demonstrated that the aberrant activity of G9a increases its repressive mark by demethylating histone 3 lysine 9 (H3K9me2), thereby triggering pathological outcomes that lead to cognitive and behavioural deficits [17,18]. Remarkably, compelling evidence links G9a to the dysregulation of cellular autophagy, repressing genes associated with autophagosome formation [19].

The autophagic process is the primary pathway for degrading defective proteins and organelles, and is orchestrated by autophagy-related proteins, such as Beclin-1, microtubule-associated protein light chain 3B (LC3B) and p62, among others. Impaired autophagy and dysregulation of the autophagic machinery have been observed in AD patients, with a resulting accumulation of toxic proteins, such as amyloid β [20,21]. Likewise, recent data indicate that autophagy deficiencies correlate with cognitive and behavioural impairments [22]. Furthermore, exciting findings show that autophagy is also involved in maintaining the integrity of synaptic vesicle-dependent transmitter release and organelle quality control, as well as in the protein homeostasis of synaptic proteins at postsynaptic sites [23,24]. In AD patients, synaptic loss is an early critical event intimately linked to cognitive decline and BPSD [25,26].

The above background supports our hypothesis that epigenetic enzymes, especially G9a, might guide cognitive and behavioural symptoms in dementia and AD. Given that sporadic late-onset AD (LOAD) accounts for most human AD cases [27], we used a SAMP8 model of age-related neurodegeneration underlying LOAD [10]. This model, established through phenotypic selection from AKR/J mice, presents AD-like cognitive symptoms and behavioural abnormalities, including anxiety-like behaviour and depression [28,29]. Additionally, SAMP8 brains are characterized by pathological signatures of AD, including neuroinflammation, synaptic deficits, oxidative stress and aberrant epigenetic dysregulation [8,9,11,30]. Thus, we evaluated the behavioural and cognitive states of aged male SAMP8 mice and determined the molecular changes in G9a-related autophagic and synaptic plasticity markers compared with senescence-accelerated mouse resistant mice (SAMR1), a healthy control strain. Our results provide insights for building the relationship between these parameters during age-related cognitive decline. 

## 2. Materials and Methods

### 2.1. Animals

Male SAMP8 (n = 13) and SAMR1 (n = 13) mice aged 12 months were used to perform behavioural experiments. Animals had free access to food and water, and were maintained under standard-temperature conditions (22 ± 2 °C), with a relative air humidity of about 50% and 12 h light-dark cycle (300 lux/0 lux), starting at 8 AM in plastic cages with wood shavings as bedding and four animals per cage. After weaning, 4–5 mice were housed per cage and behavioural tests were performed during the light phase. The mice completed a behavioural test battery at 12 months of age and were tested in the elevated plus-maze (EPM), open field (OF), novel object-recognition (NORT) and three-chamber (TCT) tests, followed by resident intruder (RI) testing. The mice were handled only during weekly cage changes before the tasks and were acclimated (5 min) to the testing room before the start of each behavioural task. Furthermore, there was a rest day between different tests.

All experimental procedures involving animals were in line with the Directive 2010/63/EU and were approved by the Institutional Animal Care and Generalitat de Catalunya (#10291, 28 January 2018). All efforts were made to minimize the number of mice used and their suffering.

### 2.2. Behavioural Experiments

#### 2.2.1. Elevated Plus Maze (EPM)

The EPM assessed the anxiety-like behaviour in an apparatus that consisted of two open arms (30 × 5 × 15 cm) and two closed arms (30 × 5 × 15 cm) radiating from a central platform (5 × 5 cm). Behaviour was scored as previously described [31]. The parameters recorded included the total distance travelled during the 5 min test, time spent in the open arms, closed arms and centre, as well as the number of rearings. 

#### 2.2.2. Open Field Test (OF)

Emotional alterations were evaluated by the OF test performed using a white polywood apparatus (50 × 50 × 25 cm), as previously described [31]. The apparatus’ ground was divided into central and peripheral areas. Mice were placed at the centre of the open field arena and allowed to explore the apparatus for 5 min. After the session, the mice were returned to their home cages, and the open field was cleaned with 70% EtOH and allowed to dry between sessions. The parameters scored included locomotor activity, calculated as the sum of total distance travelled in 5 min, as well as the time spent in the centre and periphery zone, and the number of rearings, defecations and urinations. 

#### 2.2.3. Novel Object-Recognition Test (NORT)

As previously described, the NORT was performed in a 90-degree, two-arm, 25 cm-long and 20 cm-high maze [32]. The walls could be easily removed for cleaning. Briefly, the mice were individually habituated to the apparatus for 10 min for 3 consecutive days. On day 4, they were submitted to a 10 min acquisition trial (familiarization phase), during which they were placed in the maze and allowed to explore two identical novel objects (A + A or B + B) placed at the end of each arm. Ten-minute retention trials (test phase) occurred 2 h after the familiarization phase. During the test phase, one of the two identical objects was replaced by a novel one and the time spent exploring the new object (TN) and the old object (TO) was measured manually. The discrimination index (DI) was calculated as (TN − TO/TN + TO). 

#### 2.2.4. Three-Chamber Test (TCT)

The TCT assesses cognition through sociability and interest in social novelty [33]. The TCT was performed using a box (15 × 15 × 20 cm) divided into three equally dimensioned rooms with openings between them. The testing duration was 20 min, and the test occurred in two phases within the TCT apparatus. The animal was placed in the centre of the box and allowed to explore the three chambers for 5 min (Habituation phase). The time spent in each room during the habituation phase was measured manually. Then, an intruder (same sex and age) was introduced to one chamber in a metal cage, and the behaviour of the test subject was assessed for 10 min. In this phase, both the time spent in each room and the time interacting with the intruder (e.g., sniffing and grooming) were measured. The TCT apparatus, the surface and the metal cages were cleaned with 70% EtOH between the animals’ trials to eliminate olfactory cues.

#### 2.2.5. Resident-Intruder Test (RI)

The RI test was performed to evaluate the aggressive behaviour exhibited by the animals, as described previously [34]. Briefly, the test subjects (residents) were isolated in separate cases for 7 days prior to the performance of the test. An intruder animal (C57/BL6 male, 2 months old) was introduced to the resident home cage on the test day. The entire 20 min session was video-recorded for later analysis. The aggressive behaviour of the residents was evaluated as latency to the first attack, offensive uprights, lateral threats, total number of attacks and the overall time of aggression (%), which was measured manually. In addition, social interaction during the 20 min session was evaluated as the number of rearings. 

### 2.3. RNA Extraction and Gene Expression Determination

Total RNA isolation from brain tissue (n = 6 mice per group) was carried out using TRIsureTM reagent following the manufacturer’s instructions (Bioline Reagent, London, UK). The RNA content in the samples was measured at 260 nm, and the purity of the samples was determined by the A260/280 and A260/230 ratio in a NanoDrop™ ND-1000 (Thermo Scientific, Waltham, MA, USA). The reverse transcription–polymerase chain reaction (RT-PCR) was performed as previously described [32] to quantify the mRNA expression. The primers are listed in Table S1. Data were analysed by using the comparative cycle threshold (Ct) method (ΔΔCt), using the β-actin transcript level to normalize differences. Each sample (n = 4–6) was analysed in duplicate, and the results represented the n-fold difference in the transcript levels between different samples. 

### 2.4. Western Blot (WB)

For WB, brain tissues (n = 6 mice per group) were homogenized with lysis buffer containing phosphatase and protease inhibitors (Cocktail II, Sigma, St. Louis, MI, USA), and Bradford’s method was used to determine the protein concentration. Aliquots of 15 µg of protein were separated by SDS-PAGE (10–16% gels) and transferred into a PVDF membrane (Millipore, Burlington, MA). Afterwards, the membranes were blocked with 5% BSA for 1 h at room temperature. The blockage was followed by overnight incubation at 4 °C with the primary antibodies (Table S2). The next day, the membranes were washed and incubated with secondary anti-mouse or anti-rabbit antibody for 1 h at room temperature (Table S2). The proteins were revealed using chemiluminescence-based detection kits (Thermofisher and ECL kit, Millipore, Burlington, MA, USA) and images were obtained using an Amersham Imager 680 (BioRad, Hercules, CA, USA). Then, quantitative analysis was carried out using ImageLab Software (Bioke, CA, USA) and the results were expressed in arbitrary units (AU), considering the control group as 100%. To ensure that the differences between the samples were not a result of inaccurate sample preparation, glyceraldehyde 3-phosphate dehydrogenase (GAPDH) was used as a protein charge control.

### 2.5. Dendritic Length, Spine Density and Golgi Stain Protocol

Mice brains (n = 5 per group) were removed from the skull after cervical dislocation. Then, the Golgi stain protocol was followed using an FD Rapid GolgiStain^TM^ kit according to the manufacturer’s instructions. The analysis of dendritic branching was performed with images of neurons obtained at 20× magnification using an Olympus BX61 microscope coupled to an Olympus DP70 camera. Measurements of neurite length and complexity were carried out with NeuronJ macros and Advanced Sholl Analysis. Then, the number of intersections or branch points within concentric circles with a 10 µm radius was determined and compared between groups. Analysis of spine density was conducted using the images acquired in the brightfield microscopy with a 50× oil objective lens. All analysed neurites were around 18 µm, and they were at a maximum distance of 150 µm from the soma.

### 2.6. H3 Modification Patterns Quantification

Global histone H3 methylation from brain tissue (n = 6) was detected using an EpiQuik Histone H3 Modification Multiplex Assay Kit (Catalog No. P-3100-96, Epigentek, Farmingdale, NY, USA). Briefly, the total core histones were extracted with an EpiQuik Total Histone Extraction Kit according to the manufacturer’s protocol (Epigentek, Farmingdale, NY, USA). Histone extracts were used to determine the histone H3 modifications at specific sites. The isolated histones were added to wells coated with antibodies specific for H3K9. The captured histones were detected with a detection antibody, followed by a colour-development reagent. The absorbance intensity was measured at 450 nm with a microplate reader. The amount of a particular histone’s modification was calculated as a percentage of the total H3 signal.

### 2.7. Statistical Analysis

Data are expressed as the mean ± standard error of the mean (SEM). Statistical analyses were performed using GraphPad Prism ver. 9. The normality of the data distribution was determined using the Kolmogorov–Smirnov normality test, followed by Student’s *t*-test (in the case of normal distribution) or the Mann–Whitney *U* test (in the case of non-normal distribution). Correlations between different parameters were analysed with Pearson’s and Spearman’s correlations for normally and non-normally distributed data, respectively, when it was necessary. Statistical significance was considered when the *p*-values were <0.05. Statistical outliers were discriminated using Grubbs’ test and were removed from the analysis.

## 3. Results

### 3.1. Aged SAMP8 Male Mice Exhibited Increased Aggressive and Anxiety-like Behaviours and Social Deficits

**Figure 1:**
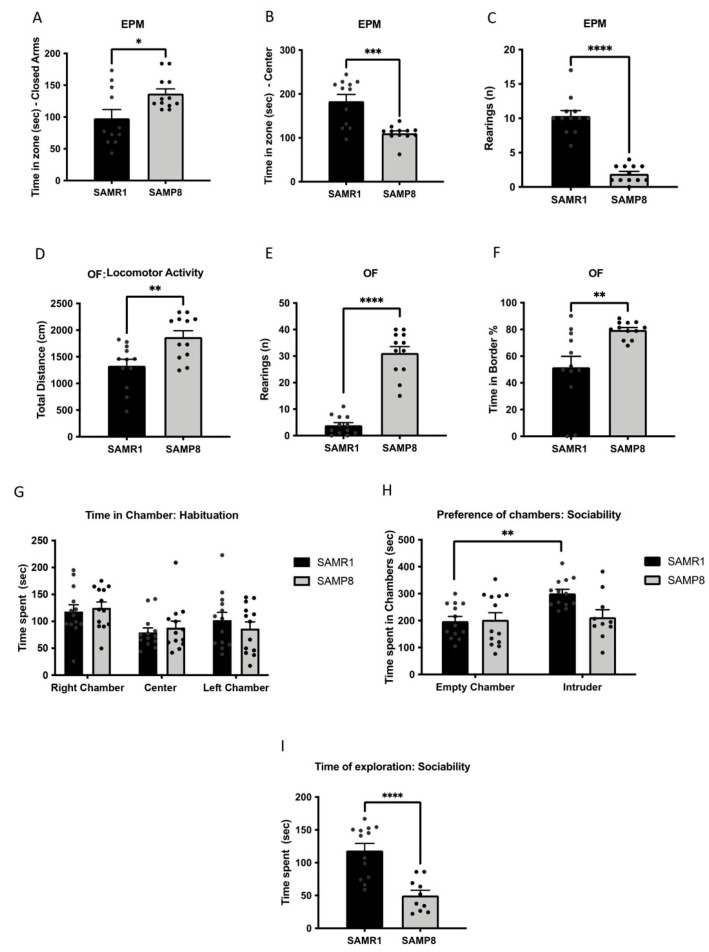
Results of the elevated plus maze (EPM) and open field (OF) tests in male SAMR1 and SAMP8 mice at 12 months of age. For EPM: time spent in closed arms (**A**), time spent in centre (**B**) and number of rearings (**C**). For OF: total distance (**D**), number of rearings (**E**) and time spent in the border zone (**F**). Results of the three-chamber test (TCT) in aged SAMR1 and SAMP8 male mice. Habituation (**G**), preference for chambers (**H**) and exploration time (**I**). Bars show the mean ± SEM (for EPM and OF: n = 11–12 mice per group; for TCT n = 13–10 mice per group). Statistical tests: t-Student analysis (**A**–**G**,**I**); two-way ANOVA followed by Šídák’s post hoc test (**H**). * *p* < 0.05; ** *p* < 0.01; *** *p* < 0.001; **** *p* < 0.0001.

Because emotional and anxiety-like behaviour are associated with cognitive decline and AD [35,36], we determined these behavioural parameters using EPM and OF. In the EPM, the SAMP8 group spent significantly more time in the closed arms (Figure 1A) and less time in the central zone compared with the SAMR1 group (Figure 1B). Furthermore, the percentage of time spent in open arms was higher in the SAMP8 group (Table S3), and the number of rearings was decreased (Figure 1C). In the OF, the SAMP8 group presented higher total distance travelled in the arena and vertical activity when compared with the SAMR1 mice (Figure 1D,E). Finally, the SAMP8 mice spent more time in the border area (Figure 1F), whereas the time spent in the central zone tended to be lower compared with that of the SAMR1 mice (Table S4).

It has been widely described that social behaviour is impaired with age and dementia [37]. To analyse this, we assessed social behaviour through TCT. Our results in the TCT revealed a relevant difference between strains in terms of sociability. Firstly, we confirmed no preference for any chamber by the two groups, since there were no differences in the time spent in each chamber during the habituation phase (Figure 1G). In the sociability phase, however, the SAMR1 group significantly spent more time in the intruder’s chamber, whereas the SAMP8 mice did not show any preference between the empty chamber and the intruder’s chamber (Figure 1H). Along the same line, the SAMP8 group spent significantly less time exploring the intruder mouse compared with the SAMR1 group (Figure 1I). 

### 3.2. Aged SAMP8 Male Mice Present Increased Aggressive Behaviour

**Figure 2:**
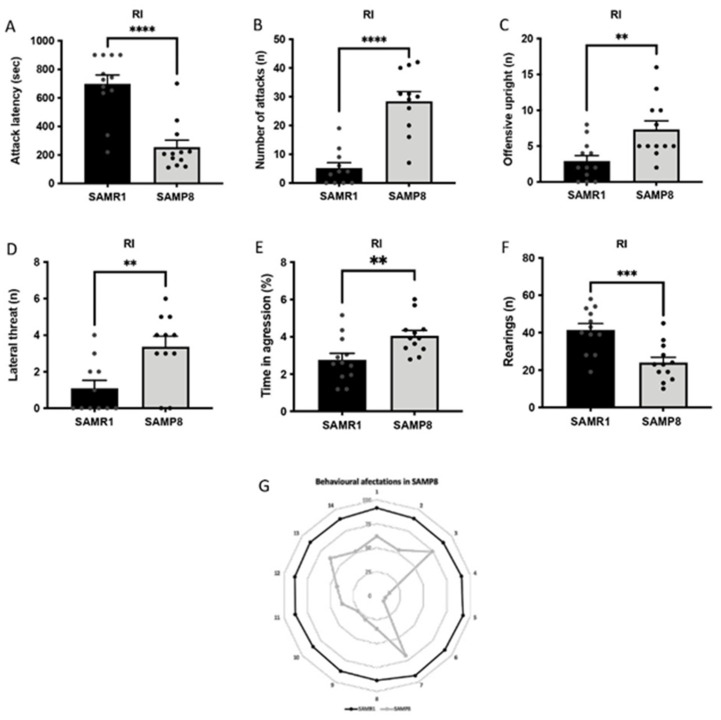
Results of the resident–intruder test (RI) in male SAMR1 and SAMP8 mice at 12 months of age. Attack latency (**A**), number of attacks (**B**), number of offensive uprights (**C**), number of lateral threats (**D**), time in aggression (**E**) and number of rearings (**F**). Bars show the mean ± SEM (For RI: n = 11–12 mice per group). Statistical test: t-Student analysis. ** *p* < 0.01; *** *p* < 0.001; **** *p* < 0.0001. Polygonal graph presenting a summary of the relevant behavioural parameters (**G**).

SAMP8 male mice showed higher aggressive behaviour compared with SAMR1 mice. In particular, the latency to the first attack against the intruder was significantly shorter for the SAMP8 mice, while the total number of aggressive encounters, including attacks and offensive uprights, was higher compared with those of the SAMR1 group (Figure 2A–C). In addition, SAMP8 mice also presented a higher number of lateral threats and percentage of time of aggression compared with age-matched SAMR1 mice (Figure 2D,E). In any case, the number of rearings was significantly reduced in the SAMP8 group, suggesting deficits in social interaction (Figure 2F). Finally, behavioural abnormalities between SAMR1 and SAMP8 mice were depicted in a polygonal graph (Figure 2G). 

### 3.3. Cognitive Deficits Correlated with Behavioural Parameters in Aged SAMP8 Male Mice

**Figure 3:**
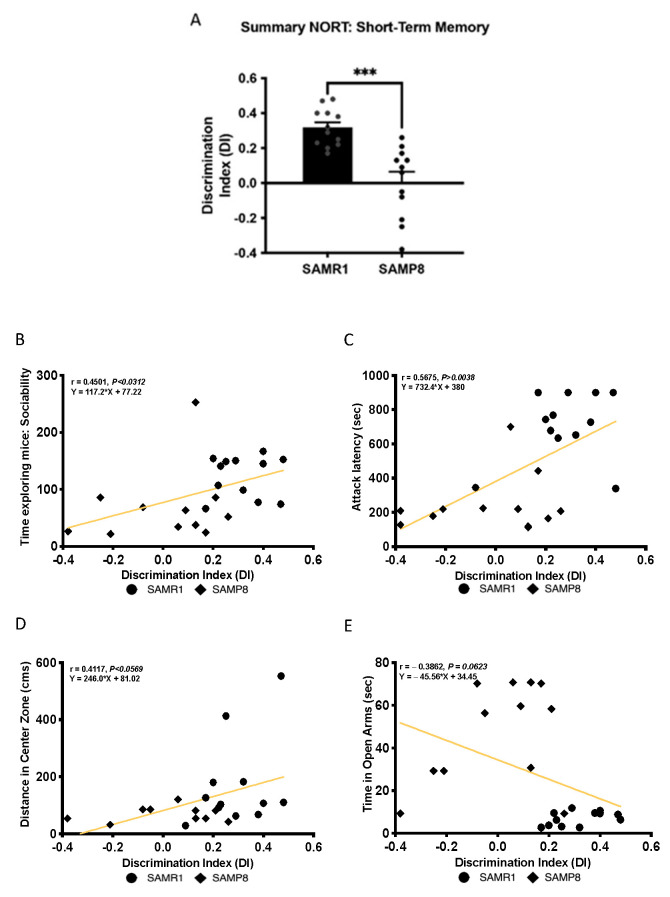
Summary of NORT for short-term memory (**A**). Bars show the mean ± SEM (for NORT: n = 12 mice per group). Statistical test: t-Student analysis. *** *p* < 0.001. Correlations between the time exploring mice indicating sociability (**B**), attack latency (**C**), distance travelled in the central zone (**D**) and time spent in open arms (**E**) and the discrimination index (DI). Pearson’s correlations were calculated for the time exploring mice (sociability), distance travelled in the central zone and time spent in open arms, and Spearman’s correlation was calculated for attack latency in both groups (n = 10–12 mice per group). r and *p*-values are indicated on the graphs.

In agreement with previous studies of our group and others [34], here, aged SAMP8 male mice presented a weaker cognitive performance than age- and sex-matched SAMR1, which resulted in significantly lower DI obtained after the 2 h short-memory test (Figure 3A). Then, we sought to determine the relationship between behaviour and cognitive parameters. First, we found a significant positive Pearson’s correlation between sociability, expressed as time exploring the intruder mouse in TCT, and the DI evaluated in NORT (r = 0.4501, *p* = 0.0312), showing that the lower social interaction of SAMP8 is associated with cognitive decline (Figure 3B). Likewise, we showed a significant positive Spearman’s correlation between the attack latency of the mice in the RI test and the DI (r = 0.5675, *p* = 0.0038), suggesting that higher aggressive behaviour exhibited by aged SAMP8 mice was related to the cognitive impairment (Figure 3C). Regarding the anxiety state of the mice, as assessed with OF and EPM, a positive Pearson’s correlation between the distance travelled in the centre zone and the DI was determined (r = 0.4117, *p* = 0.0569), whereas the time spent in the open arms presented a negative Pearson’s correlation with the DI (r = −0.3862, *p* = 0.0623). These latter correlations suggest that emotional disturbances of SAMP8 mice related to anxiety are associated with cognitive capabilities (Figure 3D,E).

### 3.4. Upregulation of G9a Correlated with the Suppression of the JNK-Mediated Autophagy Process in Aged SAMP8 Mice

**Figure 4:**
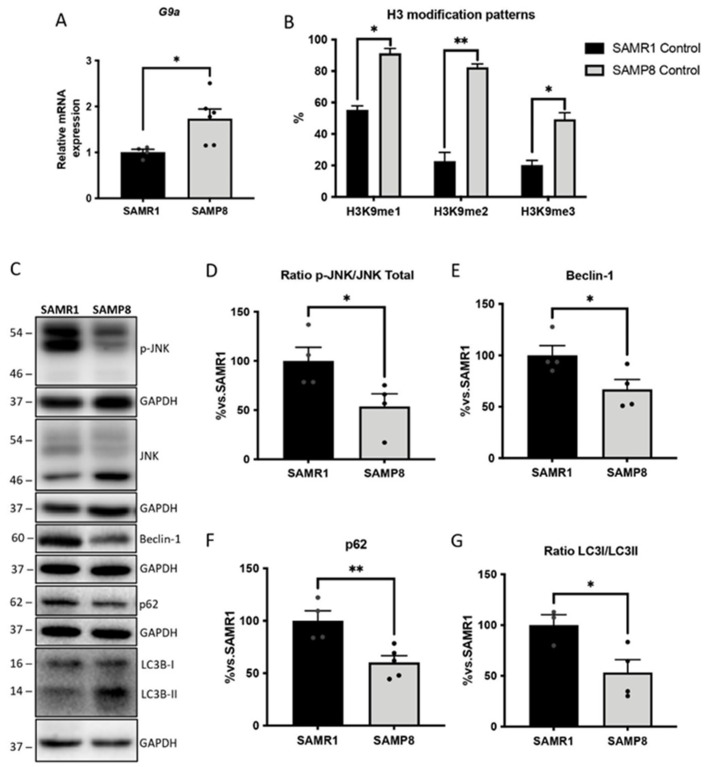
Representative gene expression for *Ehmt2* (**A**). Gene expression levels were determined by real-time PCR. Representative global H3 modification patterns’ quantification (**B**). Bars show the mean ± SEM (n = 4–6 mice per group); * *p* < 0.05; ** *p* < 0.01. Representative WBs (**C**) and quantification for the ratio of p-JNK/JNK total (**D**), Beclin-1 (**E**), p-62 (**F**) and the ratio of LC3I/LC3II (**G**). Values in the bar graphs are adjusted to 100% for the protein levels of the SAMR1 group. Bars show the mean ± SEM (n = 3–5 mice per group). Statistical test: t-Student analysis. * *p* < 0.05; ** *p* < 0.01.

G9a activity regulates autophagy in various pathological conditions [38,39]. Thus, we set out to investigate the potential epigenetic regulation of this process by G9a in aged SAMP8 mice. Our results demonstrated increased gene expression of *G9a* (*Ehmt2*) in the brains of SAMP8 mice compared with their SAMR1 counterparts (Figure 4A). As G9a is an HTM enzyme responsible for depositing methyl groups in H3K9, we also evaluated the H3 patterns. H3K9 methylated marks, including me1, me2 and me3 were found to be upregulated in SAMP8 mice (Figure 4B). Interestingly, in the neurodegenerative landscape provided by the SAMP8 mice, G9/Ehmt2 overexpression was associated with deficits in the autophagic process. In particular, the phosphorylated and, thus, activated Jun N-terminal kinase (JNK) levels were significantly lower in the SAMP8 mice compared with those of the SAMR1 strain (Figure 4C,D). Remarkably, JNK downregulation in SAMP8 brains runs parallel with decreases in key autophagic markers, including the Beclin-1, p62 and LC3B-II levels, compared with the age-matched SAMR1 mice (Figure 4C,E–G).

### 3.5. ERK Activity and Its Downstream Gene Targets in Memory Consolidation Are Downregulated in Aged SAMP8 Mice

**Figure 5:**
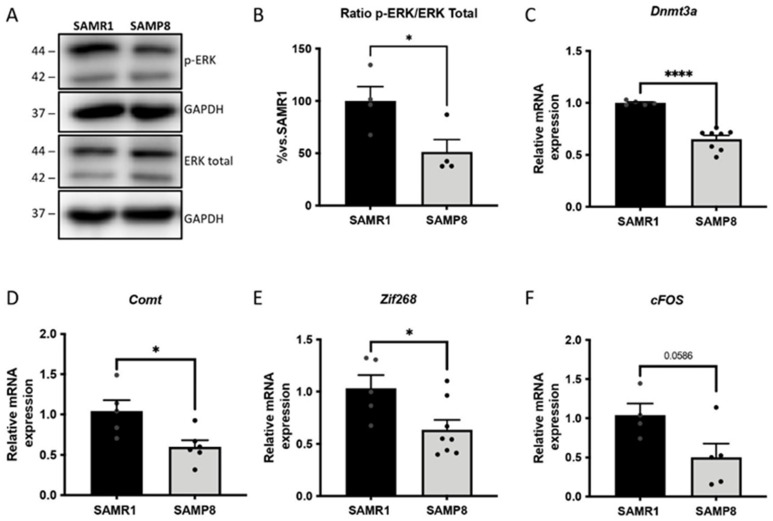
Representative WB (**A**) and quantification for the ratio of p-ERK/ERK total (**B**). Values in the bar graphs are adjusted to 100% for the protein levels of the SAMR1 group. Bars show the mean ± SEM; * *p* < 0.05. Representative gene expression levels of *Dnmt3a* (**C**), *Comt* (**D**), *Zif-268* (**E**) and *cFos* (**F**). Gene expression levels were determined by real-time PCR. Bars show the mean ± SEM (n = 4–8 mice per group). Statistical test: t-Student analysis. * *p* < 0.05; **** *p* < 0.0001.

The activation of the extracellular signal-regulated kinase mitogen-activated protein kinase (ERK MAPK) signalling cascade was evaluated in the brains of aged SAMP8 male mice. The ERK pathway plays a crucial role in the induction of neurotrophic factors and immediate early genes (IEGs) [40,41]. As expected, the results showed decreased phosphorylation (p-ERK) levels of ERK in the aged SAMP8 male mice compared with the SAMR1 group (Figure 5A,B). Furthermore, in agreement with the modulation of the ERK signalling pathway, the expression of genes modulated by ERK and involved in memory consolidation, including *DNA Methyltransferase 3 alpha* (*Dnmt3a*), *catechol-O-Methyltransferase* (*Comt*), *Zif-268* and *cFos*, was found to be reduced in the SAMP8 group compared with age-matched SAMR1 mice (Figure 5C–F).

### 3.6. Dendritic Morphological Abnormalities Accompanied Reduced Expression of Synaptic Plasticity-Related Genes in Aged SAMP8 Male Mice

**Figure 6:**
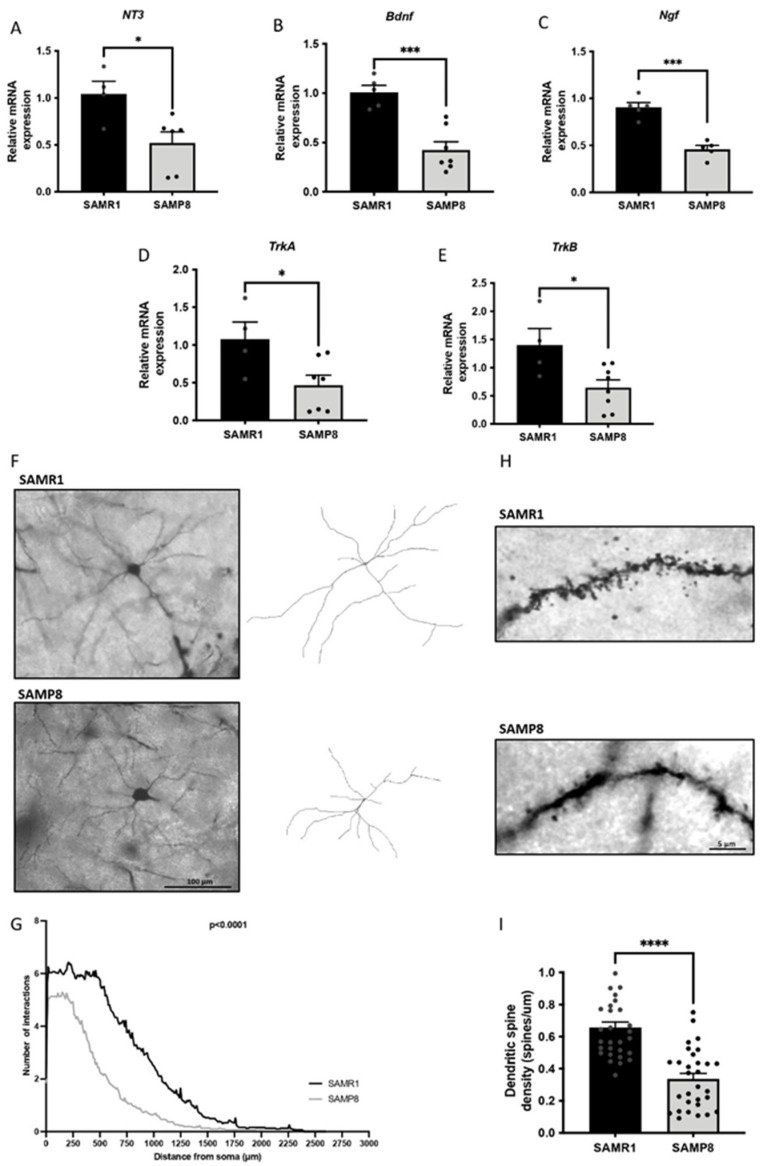
Representative gene expressions for *NT3* (**A**), *Bdnf* (**B**), *Ngf* (**C**), *TrkA* (**D**) and *TrkB* (**E**). The gene expression levels were determined by real-time PCR. Bars show the mean ± SEM (n = 4–8 mice per group). Statistical test: t-Student analysis. * *p* < 0.05; *** *p* < 0.001. Representative images and tracings of Golgi-stained cortical neurons from the aged SAMR1 male mice group (top) and the age SAMP8 male mice group (bottom) (scale bar = 100 µm) (**F**). Aged SAMP8 male mice showed a reduction in the number of neuronal intersections compared with the SAMR1 control group through a Sholl analysis (**G**). Representative images of the spine density of the aged SAMR1 male mice group (top) and the aged SAMP8 male mice group (bottom) by Golgi staining (**H**) and quantification (**I**). Bars show the mean ± SEM (n= 28–30 neurons from 6 different mice per group). Statistical test: t-Student analysis. **** *p* < 0.0001.

The activation of the ERK signalling cascade promotes the expression of neurotrophic factors [41]. Thus, we evaluated some important neurotrophins, such as brain-derived neurotrophic factor (BDNF), *neurotrophin 3* (*NT3*) and nerve growth factor (NGF) [42,43]. Strikingly, we observed a significant reduction in *NT3*, *Bdnf* and *Ngf* gene expression in the aged SAMP8 group in comparison with the SAMR1 strain (Figure 6A–C). Likewise, we evaluated the gene expression of their receptors, and we determined that the *tropomyosin receptor kinase A* and *B* (*TrkA* and *TrkB,* respectively) gene expressions were significantly lower in SAMP8 mice compared with age-matched SAMR1 mice (Figure 6D,E). Furthermore, we stained SAMP8 and SAMR1 brains using Golgi immersion to quantify neurite arborization through Sholl analysis. The results showed that the SAMP8 strain presented reduced dendritic branching compared with the SAMR1 strain (Figure 6F,G). Furthermore, we also evaluated spine density in the same vein, observing lower levels in the SAMP8 group than in the SAMR1 group (Figure 6H,I).

## 4. Discussion

Most AD patients associate cognitive dysfunction with BPSD [44]. Furthermore, about 95% of AD patients have at least one neuropsychiatric symptom and syndromes, with aggression (76.2%) and depression (68%) being the most frequent [44]. However, few studies have evaluated these symptoms unique to human beings [45] due to the lack of suitable animal models that can be directly used to study BPSD and age-related emotional alterations. At the same time, a better understanding of the molecular mechanisms underlying cognitive and non-cognitive symptoms in patients could lead to more accurate pharmacological management. Here, we assessed behavioural and emotional alterations in aged SAMP8 mice using several tasks to deeper examine the cognitive and BPSD-like phenotype of the strain. Furthermore, we established the correlation between behavioural and cognitive deficits and epigenetic alterations in the SAMP8 model. 

Enhanced fear and anxiety-like behaviour are frequently associated with ageing and AD in rodents [46,47]. Evidence shows that SAMP8 mice present an age-associated emotional disorder characterized by anxiety-like behaviour, which was reported to be either increased or decreased in different studies [48,49,50]. Accordingly, we obtained mixed results when the OF and EPM tests were employed. On the one hand, SAMP8 mice spent more time in the OF border zone and slightly less time in the centre zone, and presented a higher locomotor activity and number of rearings, demonstrating a thigmotaxis attitude and corroborating the higher anxiety-like behaviour observed in the SAMP8 strain. On the other hand, SAMP8 spent significantly more time in the open arms of the EPM and less time in the centre of the maze, which could indicate lower anxiety-like behaviour levels in the SAMP8 mice. However, data from the same behavioural test indicated that SAMP8 mice also tended to spend more time in the closed arms of the maze when compared with the SAMR1 mice. These two latter observations suggest that the parallel increases in the time spent in both open and closed maze arms could be due to the higher locomotor activity that SAMP8 showed, making the EPM data interpretation unclear. More importantly, fear and anxiety-like behaviour have been linked to age-related cognitive deficits in SAMP8 mice [51,52]. Accordingly, we found a potential relationship between anxiety-related parameters and cognitive performance, indicating the possible association of emotional disturbances with memory impairment in aged male SAMP8 mice. 

Social dysfunction observed in human AD patients has been observed in transgenic AD [46,53]. In our study, 12-month-old male SAMP8 mice presented no difference in chamber preference and decreased social interaction, indicating social behaviour deficits. These findings align with previously published results describing reduced sociability in SAMP8 mice [33,51]. Jointly with social impairment, aggression is one of the most distressing consequences of dementia and is of special significance, given its direct influence on social functionality [54]. However, the evaluation of the aggressive behaviour in old SAMP8 male mice has not yet been reported, to our knowledge. Here, we demonstrated that old SAMP8 males displayed higher aggressive behaviour with lower latency to attack and a higher total number of attacks. These results add to the diverse BPSD phenotype of aged SAMP8 mice and the presence of aggressive disturbances, and corroborate the manifestation of aggression in advanced age and dementia in this mouse model. Ultimately, when investigating the relationship between social or aggressive behaviour with cognitive performance, we found significant correlations. Social interaction was positively correlated with cognitive performance, while a significant negative correlation was calculated between aggression and cognitive performance. Consequently, our findings for SAMP8 mice support the consensus that aggression and social impairment in AD and age-related dementias are associated with the degree of cognitive impairment. 

The deregulation of epigenetic mechanisms is observed during ageing, contributing to progressive deteriorations in cognitive and behavioural status [55]. Indeed, evidence supports the involvement of epigenetic mechanisms in the clinical manifestation of neurodegenerative and neuropsychiatric disorders [56,57]. In any case, in vivo animal studies have shown that transcriptional dysregulation mediated, among others, by epigenetic mechanisms guides the manifestation of many psychiatric disorders [58,59,60,61], thereby suggesting the implication of epigenetic regulation in AD-associated BPSD. In the present study, considering that histone methylation is one of the most critical modifications, we evaluated the changes in the methylation of H3K9 mediated by the methyltransferase G9a, which is linked to gene silencing [62]. Our results showed higher levels of *G9a* gene expression in the aged SAMP8 group, which was correlated with an increase in the overall H3 methylation landscape. The increased histone methylation in the brain of aged SAMP8 mice was in accordance with previous studies performed in familial AD mice, demonstrating similar increases in the H3K9me2 levels in the brain [63]. Similarly, higher levels of H3K9me2 were also described in the CA1 of AD patients compared with middle-aged control patients [64]. It is important to note that G9a, as a regulator of chromatin structure in the CNS, has been associated with brain development, the memory consolidation process and cognitive deficits [18,65]. Interestingly, G9a inhibition has been shown to improve cognitive performance in AD models [17,63], improving anxiety-like behaviour in adult mice [66]. This evidence suggests that increased G9a protein levels promoted epigenetic alterations in the SAMP8 brains that, in turn, may account for behavioural abnormalities and age-related cognitive decline. 

In light of these findings, we then investigated alterations in G9a-regulated molecular pathways in the brain of SAMP8 mice. A well-described mechanism by which G9a contributes to neurodegenerative disorders is autophagy repression [19]. It is widely accepted that the balance between protein synthesis and protein turnover is necessary for synaptic plasticity, and that this imbalance could be caused by the deregulation of autophagic activity [67]. Previous studies described the binding of G9a within the promoters of core autophagy-associated genes, compromising their activity [19]. Interestingly, the genetic depletion or pharmacologic inhibition of G9a promoted its dissociation from those inducing LC3BII and p62 expression, fostering autophagosome-like structure formation [19]. However, ChIP experiments demonstrated that the loss of G9a and H3K9me2 at the LC3B and p62 promoters was dependent on JNK activity [19]. In line with these findings, lower protein levels of JNK were observed in the aged SAMP8 group, and all of the autophagic-related proteins evaluated were downregulated. Thus, increased levels of G9a might be partially responsible for the deregulation of the JNK pathway in the aged SAMP8, leading to autophagic activity deficits, which in turn correlate with the impaired cognitive and behavioural state of SAMP8 mice. 

Moreover, it has been described that BDNF, NT3 and NGF neurotrophins are involved in BPSD, such as anxiety, depression and aggressive behaviour [68]. Here, we demonstrated a reduction in *Bdnf, NT3* and *Ngf* gene expression in aged SAMP8 male mice. In line with these results, cumulative studies with AD animal models showed that lower BDNF and NGF levels led to neuronal and synaptic dysfunction and eventual cognitive impairment [69,70,71,72]. Interestingly, BDNF transcription can be regulated by epigenetics, and G9a inhibition was shown to increase *Bdnf* expression in AD mice models [18,73]. As mentioned before, G9a has been implicated in memory consolidation mechanisms [65]. Here, we also found lower expression of the genes involved in the ERK-modulated memory consolidation process in aged SAMP8, suggesting the participation of G9a. It is also worth mentioning that *Bdnf* expression regulates and is regulated by Erk ½, further supporting the obtained results [74]. In parallel, a remarkable downregulation in the gene expression of *Trk*A and *Trk*B neurotrophin receptors was determined in aged SAMP8 mice, corroborating the deficits in the synaptic function of the SAMP8 strain. 

Along with the loss of synapses in the aged brain [75], the dendritic spine density is greatly diminished in AD, and this event is one of the most distinctive features of AD that best correlates with cognitive impairment [76,77]. Remarkably, altered dendritic spine density has been reported in models of other neurological conditions, such as depression, stress and aggressive behaviours [78,79,80,81]. Hence, dendritic arborization and spine density are vital for assessing cognitive function and behavioural alterations. As expected, we reported loss of a dendritic spines in the aged cortexes of SAMP8 mice and reduced dendritic length compared with the aged SAMR1 group. Interestingly, these results align with a previous study in which G9a inhibition promoted the improvement of the structural plasticity of the dendritic spines of neurons in the nucleus accumbens [82]. 

## 5. Conclusions

Our findings suggest that autophagic and synaptic plasticity impairments in aged male SAMP8 mice might be partially attributable to the epigenetic modulation by G9a, encouraging further pharmacological studies to establish a causal relationship between them in SAMP8 mice. Furthermore, we demonstrated that the cognitive alterations were joined with morphological modifications in neurons, and altogether could account for non-cognitive and cognitive alterations exhibited by the mouse model. Additionally, our results agree with previous findings regarding the SAMP8 mouse model and give further support to this senescence model as a gerontological research tool offering new insight into the suitability of SAM strains for the study of non-cognitive alterations accompanied by cognitive impairment of senescence.

## Data Availability

Not applicable.

## Supplementary Materials

The following supporting information can be downloaded at: https://www.mdpi.com/article/10.3390/cells11162603/s1, Table S1. Primers used in qPCR studies; Table S2. Antibodies used in the Western blot studies; Table S3. Parameters measured in the elevated plus maze (EPM) test for male SAMR1 and SAMP8 mice at 12 months of age; Table S4. Parameters measured in the open field test (OF) for male SAMR1 and SAMP8 mice at 12 months of age.
