# Supplementary material for: Cognitive Decline and BPSD Are Concomitant with Autophagic and Synaptic Deficits Associated with G9a Alterations in Aged SAMP8 Mice
Source: Cells. 2022 Aug 21;11(16):2603. doi: 10.3390/cells11162603 (PMC9406492; doi:10.3390/cells11162603)
Supplement: Supplementary file 1 [file cells-11-02603-s001.zip › Table S1.pdf]

Table S1. Primers used in qPCR studies.

Syber Green Primers

Target	Forward primer (5'-3')	Reverse primer (5'-3')
NT3	CAGGGTGAAGGGGAAAACTC	AGTTCGGTCATTCAGTCTCGC
G9a	CCAGAGGAGTGAATGGTGT	CTTTCGGTGGCCATACACTT
Dnmt3a	ACGCCAAAGAAGTGTCTGCT	CTTTGCCCTGCTTTATGGAG
Comt	CCCTCCTGTCGGATTACTCA	GGGGGAAGCACATGAGTCTA
Zif-268	TCAGCCTAGTCAGTGGCCTT	AGGTCTCCCTGTTGTTGTGG
cFos	CCCGTAGACCTAGGGAGGAC	CAATACACTCCATGCGGTTG
Bdnf	TGCGAGTATTACCTCCGCCAT	TCACGTGCTCAAAAGTGTCAG
Ngf	GGAGCGCATCGAGTGACTT	CCTCACTGCGGCCAGTATAG
TrkA	CTCCTTCTCGCCAGTGGAC	TGCCCTCAGTAGGGGAAAGA
TrkB	CGTCACTTCGCCAGCAGTAG	CTATACGCCAGGCACCACTC
β-Actin	CAACGAGCGGTTCCGAT	GCCACAGGTTCCATACCCA
