# Supplementary material for: Cognitive Decline and BPSD Are Concomitant with Autophagic and Synaptic Deficits Associated with G9a Alterations in Aged SAMP8 Mice
Source: Cells. 2022 Aug 21;11(16):2603. doi: 10.3390/cells11162603 (PMC9406492; doi:10.3390/cells11162603)
Supplement: Supplementary file 1 [file cells-11-02603-s001.zip › Table S2.pdf]

Table S2. Antibodies used in Western blot studies.

Antibody	Host	Source/Catalog	WB dilution
**GAPDH**	Mouse	Millipore/MAB374	1:2000
**JNK**	Mouse	Santa Cruz/sc-7345	1:500
**p-JNK**	Rabbit	Cell Signalling/#9251	1:1000
**Beclin-1**	Rabbit	Cell Signalling/#3738	1:1000
**p-62**	Mouse	Santa Cruz/sc-28359	1:500
**LC3B**	Rabbit	Cell Signalling/2775	1:1000
**ERK**	Rabbit	Cell Signalling/9102	1:1000
**p-ERK**	Rabbit	Cell Signalling/9101	1:1000
**Goat-anti-mouse HRP conjugated**		BioRad/170-5047	1:2000
**Goat-anti-rabbit HRP conjugated**		BioRad/170-6515	1:2000
