# Supplementary material for: Cognitive Decline and BPSD Are Concomitant with Autophagic and Synaptic Deficits Associated with G9a Alterations in Aged SAMP8 Mice
Source: Cells. 2022 Aug 21;11(16):2603. doi: 10.3390/cells11162603 (PMC9406492; doi:10.3390/cells11162603)
Supplement: Supplementary file 1 [file cells-11-02603-s001.zip › Table S3.pdf]

Table S3. Parameters measured in the Elevated Plus Maze (EPM) in male SAMR1 and SAMP8 mice at 12 months of age. (n): number of events. Results are expressed as a mean ± Standard error of the mean (SEM). ***p<0.001; ****p<0.0001.

	**SAMR1**	**SAMP8**
**Locomotor activity (cm)**	789.55 ± 44.21	892.42 ± 39.05
**Rearings (n)**	10.33 ± 0.79	1.92 ± 0.36 ****
**Time in Zone (%)- Center**	61.21 ± 5.05	36.89 ± 1.74 ***
**Time in Zone (%)- Open Arms**	2.92 ± 0.56	17.06 ± 2.86 ****
**Time in Zone (%)- Closed Arms**	35.54 ± 5.04	45.63 ± 2.52
