# Supplementary material for: Cognitive Decline and BPSD Are Concomitant with Autophagic and Synaptic Deficits Associated with G9a Alterations in Aged SAMP8 Mice
Source: Cells. 2022 Aug 21;11(16):2603. doi: 10.3390/cells11162603 (PMC9406492; doi:10.3390/cells11162603)
Supplement: Supplementary file 1 [file cells-11-02603-s001.zip › Table S4.pdf]

Table S4. Parameters measured in the Open Field Test (OFT) in male SAMR1 and SAMP8 mice at 12 months of age. (n): number of events. Results are expressed as a mean ± Standard error of the mean (SEM). *p<0.05; **p<0.01; ****p<0.0001.

	**SAMR1**	**SAMP8**
**Locomotor activity (cm)**	1476.18 ± 85.68	1870.07 ± 118.8 *
**Rearings (n)**	4.7 ± 1.08	31.17 ± 2.41 ****
**Time in Zone (%)- Border**	61.93 ± 5.42	79.63 ± 1.83 **
